# Impact of quetiapine on resolution of individual delirium symptoms in critically ill patients with delirium: a *post-hoc *analysis of a double-blind, randomized, placebo-controlled study

**DOI:** 10.1186/cc10450

**Published:** 2011-09-17

**Authors:** John W Devlin, Yoanna Skrobik, Richard R Riker, Eric Hinderleider, Russel J Roberts, Jeffrey J Fong, Robin Ruthazer, Nicholas S Hill, Erik Garpestad

**Affiliations:** 1 Northeastern University School of Pharmacy, 360 Huntington Avenue, Mugar 206, Boston, MA 02115, USA; 2 Division of Pulmonary, Critical Care and Sleep Medicine, Tufts Medical Center, 800 Washington Street, Boston, MA 02111, USA; 3 Department of Critical Care Medicine, Maisoneuve-Rosemont Hospital, 5415 de l'Assomption, Montreal, QC H1T 2M4, Canada; 4 Department of Critical Care Medicine, Maine Medical Center, 22 Bramhall Street, Portland, ME, 04102, USA; 5 Department of Pharmacy, Tufts Medical Center, 800 Washington Street, mailstop #420, Boston, MA 02111, USA; 6 Department of Pharmacy Practice, Massachusetts College of Pharmacy and Health Sciences, 19 Foster Street, Worcester, MA 01608, USA; 7 Institute for Clinical Research and Health Policy Studies, Biostatics Research Center Tufts Medical Center, 35 Kneeland Street, Boston, MA 02111, USA

## Abstract

**Introduction:**

We hypothesized that delirium symptoms may respond differently to antipsychotic therapy. The purpose of this paper was to retrospectively compare duration and time to first resolution of individual delirium symptoms from the database of a randomized, double-blind, placebo-controlled study comparing quetiapine (Q) or placebo (P), both with haloperidol rescue, for critically ill patients with delirium.

**Methods:**

Data for 10 delirium symptoms from the eight-domain, intensive care delirium screening checklist (ICDSC) previously collected every 12 hours were extracted for 29 study patients. Data between the Q and P groups were compared using a cut-off *P-*value of ≤0.10 for this exploratory study.

**Results:**

Baseline ICDSC scores (5 (4 to 7) (Q) vs 5 (4 to 6)) (median, interquartile range (IQR)) and % of patients with each ICDSC symptom were similar in the two groups (all *P *> 0.10). Among patients with the delirium symptom at baseline, use of Q may lead to a shorter time (days) to first resolution of symptom fluctuation (4 (Q) vs. 14, *P *= 0.004), inattention (3 vs. 8, *P = *.10) and disorientation (2 vs. 10, *P *= 0.10) but a longer time to first resolution of agitation (3 vs. 1, *P *= 0.04) and hyperactivity (5 vs. 1, *P *= 0.07). Among all patients, Q-treated patients tended to spend a smaller percent of time with inattention (47 (0 to 67) vs. 78 (43 to 100), *P *= 0.025), hallucinations (0 (0 to 17) vs. 28 (0 to 43), *P *= 0.10) and symptom fluctuation (47 (19 to 67) vs. 89 (33 to 00), *P *= 0.04] and there was a trend for Q-treated patients to spend a greater percent of time at an appropriate level of consciousness (26% (13 to 63%) vs. 14% (0 to 33%), *P *= 0.17].

**Conclusions:**

Our exploratory analysis suggests that quetiapine may resolve several intensive care unit (ICU) delirium symptoms faster than the placebo. Individual symptom resolution appears to differ in association with the pharmacologic intervention (that is, P vs Q, both with as needed haloperidol). Future studies evaluating antipsychotics in ICU patients with delirium should measure duration and resolution of individual delirium symptoms and their relation to long-term outcomes.

## Introduction

Delirium, characterized by acute disturbances of consciousness and cognition that develop over a short time in the context of an acute medical condition, occurs in up to 50% of intensive care unit (ICU) patients [1,2]. Critically ill patients who develop delirium have a higher mortality, a longer duration of mechanical ventilation and may develop long-term cognitive abnormalities [3-7]. The Intensive Care Delirium Screening Checklist (ICDSC) is an eight-domain instrument that was specifically developed and validated for screening delirium in ICU patients [8]. It has been shown to identify delirium as well as psychiatrists using Diagnostic and Statistical Method (DSM) IV criteria, to be highly reliable, to improve the ability of both ICU physicians and nurses to identify delirium in their patients and is supported by formal pedagogical testing [8-13]. In contrast to binary scales, such as the CAM-ICU, the presence of individual delirium symptom features is an inherent part of the diagnostic criteria [14]. In addition, the ICDSC can identify subsyndromal delirium, a clinically important syndrome that identifies patients that fall on a continuum between those with no neuropsychiatric symptoms and those with DSM IV-defined delirium [15-18]. Patients with subsyndromal delirium have a mortality and length of stay that lies between normal patients and those with delirium, and represents a cohort of patients especially responsive to treatment measures for delirium.

Whether the administration of antipsychotic medications improves outcome of ICU patients with delirium remains controversial [19]. Among the three published pilot prospective, randomized trials (only two of which have been placebo-controlled) that have evaluated antipsychotic pharmacotherapy to prevent or treat delirium in the ICU, only one small randomized, double-blind, placebo-controlled study has demonstrated benefit [20-22]. In this study, ICU patients with delirium who required the administration of one or more doses of IV haloperidol were randomized to either quetiapine or placebo, both with as needed rescue therapy [20]. Quetiapine (up to 200 mg twice daily for a maximum of 10 days) was associated with a shorter time to first resolution of delirium (1.0 (interquartile range (IQR), 0.5 to 3.0) vs. 4.5 days (IQR, 2.0 to 7.0; *P *= .001)) and a reduced duration of delirium (36 (IQR, 12 to 87) vs. 120 hrs (IQR, 60 to 195; *P *= .006)) [20]. In other studies, delayed treatment once delirium is established has been reported as being associated with worse outcomes [23]. Whether antipsychotic administration is beneficial for ICU delirium may depend on drug choice, but may also vary with symptom constellation and the effect of a given drug on specific symptoms (as has been shown for delirium outside the ICU setting) [24,25].

The outcome of patients with delirium has been linked to the particular delirium symptoms identified with ICDSC evaluation [26]. However, no ICU delirium prevention or treatment strategy published to date reports the impact of interventions on time to resolution of individual delirium symptoms [20-22,27-32]. We hypothesized that the pattern of delirium symptom resolution would differ between patients receiving quetiapine vs. placebo. In this *post-hoc *analysis, we compared the delirium symptoms evaluated by the ICDSC within our previous randomized study in terms of the time to first resolution and the duration of each delirium symptom [20].

## Materials and methods

Data for this IRB-approved, retrospective, *post-hoc *analysis is derived from a three-center, double-blind, placebo-controlled study that evaluated the efficacy and safety of quetiapine in adult ICU patients with delirium (ICDSC ≥4), who had an order for as-needed haloperidol and were tolerating enteral nutrition [20]. While informed consent was obtained from all subjects in the original study, the IRB did not require subjects to provide consent for their data to be used in this secondary analysis given its *post-hoc *nature. During the study period 258 ICU patients were screened and 222 were excluded. The exclusion criteria for this study were extensive and included conditions that could mimic delirium or place patients at increased risk for quetiapine-related adverse effects. Delirium assessments were conducted every 8 to 12 hours by trained bedside nurses using the ICDSC. All patients were managed with the same as-needed intravenous haloperidol protocol. Therapy (that is, quetiapine or a matching placebo) was administered orally or via a nasogastric/enteral tube and initiated at a dose of 50 mg every 12 hours. Therapy was titrated upwards on a daily basis by increments of 50 mg every 12 hours to a maximum dose of 200 mg every 12 hours if the subject received at least one dose of as-needed haloperidol in the previous 24 hours. The study drug was continued until one of the following occurred: 1) the subject was deemed by the attending intensivist (based on their clinical judgment) to no longer demonstrate signs of delirium and, therefore, to no longer require therapy with a scheduled antipsychotic; 2) 10 days of therapy had elapsed; 3) ICU discharge occurred; or 4) an adverse event potentially attributable to the study drug occurred.

The ICDSC data were extracted from the study database for subjects with more than half of the every-12-hour ICDSC assessments completed in full during blinded study drug administration. If more than one ICDSC score was available for each 12-hour study period, the ICDSC with the highest score was used. While the ICDSC evaluates eight different domains associated with delirium, it actually evaluates 10 different delirium symptoms [8]. Data from ICDSC domain #1 (level of consciousness) evaluates both agitation and decreased level of consciousness and ICDSC domain #5 (motor activity) evaluates both hyperactivity and hypoactivity. Therefore, these four symptoms for domains #1 and #5 were collected with the single symptoms from the other six domains, resulting in 10 different symptoms of delirium being evaluated.

The prevalence of each delirium symptom present at the study baseline was compared between the quetiapine and placebo groups using Pearson's chi-square tests. Among subsets of patients with each symptom at baseline, the proportion experiencing symptom resolution was compared between groups using Pearson's chi-square tests. Because not all patients with symptoms experienced resolution of symptoms, Kaplan-Meier curves were generated and used to compare the time to first symptom resolution between groups. Among all study patients, for each symptom, the median time to first resolution for each group (that is, quetiapine versus placebo) was estimated from the Kaplan-Meier curves and the Log-rank test was used to compare the curves between the two groups. For each patient the proportion of time that each symptom was present was calculated. Additionally, the percent time when patients were at an appropriate level of consciousness was determined by subtracting the sum of (agitation plus decreased level of consciousness) from 1. Because the distribution of these proportions tended to be skewed, a nonparametric Wilcoxon rank sum test was used to compare the distributions between the quetiapine and placebo groups and data were summarized as medians and interquartile ranges (25th to 75th percentiles).

Given these were exploratory analyses and the fact that the original trial was not designed or powered to find associations with individual symptoms, we selected an alpha of *P *≤0.10 as our threshold for concluding statistical significance. While this higher threshold is more likely to find between-group differences when none exist (1/10 chance) when compared to the 1/20 chance when *P *< 0.05 is used, it is also more likely to find a significant association when one does exist, reducing type II errors. No adjustments to *P*-values for multiple comparisons were made given the exploratory nature of this study and unadjusted *P*-values for every comparison done are presented in tables.

## Results

Acceptable data for the analysis of ICDSC delirium symptoms during blinded study drug administration were available for 29 of the 36 patients enrolled in the parent pilot study which required a diagnosis of delirium for enrollment (quetiapine; *n *= 14; placebo; *n *= 15). The information on the first seven patients enrolled at one of the study sites included only the ICDSC total score and the detailed individual symptoms were not available for this *post-hoc *analysis. All 29 other patients had at least 50% complete symptom data recorded and were included in this retrospective study. Overall, 16% of all possible ICDSC symptom data-points were not able to be assessed. Excessive sedation and inattention were the most common reasons for not assessing all ICDSC symptoms during the ICDSC assessments included in the analysis. Baseline age, gender, Acute Physiology and Chronic Health Evaluation (APACHE) II score and proportion of patients intubated were similar between each *post-hoc *group and the similar group in the parent study. At baseline, neither the ICDSC score (5 (4 to 6) (quetiapine) versus 5 (4 to 6); *P *= 0.32)) nor the incidence of each delirium symptom was different between the two groups (Table 1).

**Table 1:** Prevalence of delirium symptoms evaluated by the ICDSC at study baseline

Delirium symptons derived from ICSDC	Quetiapine (*n *= 14) N (%)	Placebo (*n *= 15) N (%)	*P*-value
**Agitation**	5 (35.7)	6 (40.0)	0.81
**Decreased level of consciousness**	6 (42.9)	6 (40.0)	0.88
**Inattention**	13 (92.9)	15 (100.0)	0.29
**Disorientation**	13 (92.9)	11 (84.6)	0.49
**Hallucinations/Delusions**	2 (14.3)	1 (6.7)	1.00
**Hyperactivity**	5 (35.7)	6 (40.0)	0.81
**Hypoactivity**	9 (64.3)	9 (60.0)	0.81
**Inappropriate speech or mood**	7 (77.8)	3 (42.9)	0.15
**Sleep/wake cycle disturbances**	8 (66.7)	10 (71.4)	0.79
**Symptom fluctuation**	11 (91.7)	13 (86.7)	0.68

ICDSC, Intensive Care Delirium Screening Checklist

Among patients with the delirium symptom at baseline, use of quetiapine may lead to a shorter time to first resolution of symptom fluctuation (4 (quetiapine) vs. 14 days, *P *= 0.004), inattention (3 vs. 8 days, *P *= 0.10) and disorientation (2 vs.10 days, *P *= 0.10), but a longer time to resolution of agitation (3 (quetiapine) vs. 1 days, *P *= 0.04) and hyperactivity (5 vs. 1 days, *P *< 0.04) (Table 2). Among all patients, quetiapine-treated patients tended to spend less percentage of time with inattention (47 (0 to 67) vs. 78 (43 to 100), *P *= 0.025], hallucinations (0 (0 to 17) vs. 28 (0 to 43), *P *= 0.10) and symptom fluctuation (47 (19 to 67) vs. 89 (33 to 100), *P *= 0.04) and there was a trend for quetiapine-treated patients to spend more percentage of time at an appropriate level of consciousness (26 (13 to 63) vs. 14 (0 to 33), *P *= 0.17) (Table 3).

**Table 2:** Proportion of patients experiencing resolution and time to first resolution for the delirium symptoms^A^

	**Resolved during therapy**^**A **^**N (%)**			**Time to first resolution**^**A**^ (hours; median (IQR))		
Delirium symptoms derived from ICDSC	Quetiapine (*n *= 14)	Placebo (*n *= 15)	^B^*P*-value	Quetiapine (*n *= 14)	Placebo (*n *= 15)	^C^*P*-value
Agitation	5 (100.0)	6 (100.0)	-	84 (48 to 204)	36 (24 to 60)	0.07
Decreased level of consciousness	5 (83.3)	6 (100.0)	0.30	36 (24 to 72)	24 (12 to 96)	0.78
Inattention	12 (92.3)	11 (73.3)	0.19	84 (24 to 168)	180 (72 to 240)	0.12
Disorientation	11 (85.6)	8 (72.7)	0.47	48 (12 to 216)	204 (36 to 240)	0.10
Hallucinations/delusions	2 (100.0)	1 (100.0)	-	48 (24 to 72)	156 (144 to 156)	0.22
Hyperactivity	5 (100.0)	6 (100.0)	-	120(60 to 192)	24 (12 to 48)	0.04
Hypoactivity	7 (77.8)	6 (66.7)	0.60	72 (48 to 132)	168 (96 to ne)	0.17
Inappropriate speech or mood	6 (85.7)	3 (100.0)	0.49	132(60 to 192)	72 (24 to 168)	0.31
Sleep/wake cycle disturbance	5 (62.5)	6 (60.0)	0.91	192 (24 to ne)	228 (144 to 240)	0.36
Symptom fluctuation	11 (100.0)	7 (53.8)	0.009	4 (1 to 10)	14 (7 to ne)	0.004

ICDSC, Intensive Care Delirium Screening Checklist

IQR, Interquartile range expressed as 25th to 75th percentile; the value of "ne" indicates the value could not be estimated from these

^B^*P*-value from comparison of proportion resolved between the two groups using the Pearson's Chi-square test.

^C^*P*-value from comparison of time to first resolution between the two groups from Log-Rank test comparing Kaplan-Meier curves

**Table 3:** Time spent with each delirium symptom as a proportion of time study drug was administered^A, B^

	^C^Delirium symptom ever present (Over course of study drug; N (%))			^C^Time spent with delirium symptom (% of total days of study drug; median (IQR))		
Delirium symptoms derived from ICDSC	Quetiapine (*n *= 14)	Placebo (*n *= 15)	*P*-value	Quetiapine (*n *= 14)	Placebo (*n *= 15)	^C^*P*-value
**Agitation**	6 (42.9)	7 (46.7)	0.83	0 (0 to 13)	14 (0 to 57)	0.17
**Decreased level of consciousness**	8 (57.1)	7 (46.7)	0.72	3 (0 to 50)	22 (7 to 29)	0.24
**Inattention**	13 (92.9)	15 (100)	0.29	47 (0 to 67)	78 (43 to 100)	0.03
**Disorientation**	13 (92.9)	12 (80)	0.59	35 (0 to 55)	52 (25 to 94)	0.27
**Hallucinations/delusions**	2 (14.3)	1 (6.7)	1.00	0 (0 to 17)	28 (0 to 43)	0.10
**Hyperactivity**	6 (42.9)	7 (46.7)	0.79	0 (0 to 20)	14 (0 to 57)	0.28
**Hypoactivity**	9 (64.3)	9 (60.0)	0.81	50 (17 to 83)	67 (22 to 88)	0.61
**Inappropriate speech or mood**	7 (77.8)	4 (16.0)	0.24	15 (0 to 44)	24 (0 to 60)	0.98
**Sleep/wake cycle disturbances**	9 (64.3)	10 (71.4)	0.82	58 (33 to 100)	83 (36 to 100)	0.69
**Symptom fluctuation**	11 (91.7)	14 (93.3)	0.52	47(19 to 67)	89 (33 to 100)	0.04

ICDSC, Intensive Care Delirium Screening Checklist

^A^Among all patients and, therefore, regardless of whether each delirium symptom was present at baseline.

^B^Time spent was based on the number of 12 hour shifts where the symptom was present

^C^For comparison of distributions between the two groups for time spent with delirium symptom using the Wilcoxon rank sums test

## Discussion

Recent investigations in both ICU and non-ICU populations have expanded the classification of patients with delirium from a dichotomous clinical diagnosis of delirium (that is, delirium or not), and focused instead on specific components of delirium, recognizing the heterogeneity of patients with this complex syndrome [15,16]. Recent data highlights the importance that individual symptom recognition and monitoring can have when managing delirium [33]. Individual delirium symptoms can be screened in ICU patients using a tool such as the ICDSC. Each delirium symptom evaluated with the ICDSC is highly discriminatory when distinguishing between critically ill patients with or without delirium, and after adjustment for severity of illness and coma, specific symptoms of delirium independently predict mortality in sub-syndromal delirium [26].

Our analysis is the first to measure the response of individual delirium symptoms to antipsychotic therapy in the ICU and suggests that quetiapine use is associated with more rapid resolution of many common ICU delirium symptoms when compared to a placebo, resulting in less time affected by hallucinations, inattention, symptom fluctuation and potentially less time at an undesirable level of consciousness. Although time to first resolution of agitation and hyperactivity was longer in patients receiving quetiapine, there appeared to be more time spent in a state other than agitation or psychomotor slowing, suggesting greater "normalcy" and symptom control. It remains unclear. However, with agitation and hyperactivity providing clinicians with the greatest challenge when caring for a patient with delirium and likely leading to the administration of additional sedatives or antipsychotic medications further research is needed to carefully evaluate this potential effect. While the relative sedating effects of haloperidol versus quetiapine are not well quantified, if haloperidol has the greater sedative effect, the fact that nearly twice as much "as needed" haloperidol was administered on average to the placebo group may also account for the faster resolution of agitation in this group. Identifying which symptoms are most important regarding long-term outcomes, and how best to describe their presence or absence, will require further study. Despite the well-established sedating properties of quetiapine, sleep-wake cycle abnormalities were not improved in this small sample of patients, suggesting sleep abnormalities associated with delirium may not be palliated with quetiapine.

Few other reports of quetiapine use for delirium have been published, and none were ICU-based studies. A recent, randomized, double-blind study compared delirium resolution with quetiapine or placebo treatment among hospitalized (but not ICU) medical and surgical patients and found the cognitive components recovered 83% faster in the quetiapine group, and noncognitive scores recovered 57% faster than the placebo group [33].

A number of limitations of our study must be considered. First, patients were enrolled in the parent randomized controlled study an average of six days after ICU admission - nearly twice as long after ICU admission as when delirium often first presents [14]. Study enrollment may have been delayed by our requirement that delirium be an established diagnosis and treatment with haloperidol initiated prior to consent and randomization and that adequate gut function (for example, tolerating enteral nutrition) be established. Although not well characterized, it is possible that the individual symptoms of delirium present in a patient with delirium vary with time after ICU admission [26]. Second, while a summary ICDSC score was documented for all study patients, documentation of individual delirium symptoms (from the ICDSC) was missing for 7 of the 36 patients in the parent study. It is possible that the results of this secondary analysis may have been different had all 36 patients been included although baseline demographics and the time to first resolution of delirium were similar between each *post-hoc *group (*n *= 14, *n *= 15) and the corresponding groups in the parent study (*n *= 18, *n *= 18). Some of the delirium symptoms (for example, hallucinations) may have been detected at too low of a frequency to allow for a valid analysis of their time to resolution between the two study groups.

We provide clinical information regarding the short-term response of ICU symptoms, but did not evaluate long-term follow-up after hospital discharge for these patients. Whether long-term cognitive function may be related to specific symptoms identified during the ICU stay (as it is with delirium in general) remains unknown. Since the study was initiated only in delirious patients, the effect psychotropic drugs may have on subsyndromal delirium remains unexplored. With multiple comparisons of 10 symptoms that were tested and the small sample size, we chose to look for promising differences which may serve as a foundation for future studies powered for this type of analysis. We also present the individual unadjusted *P*-values for every comparison made, in the spirit of maximal transparency and to fuel future studies in this area.

## Conclusions

Our exploratory analysis suggests that quetiapine appears to resolve many ICU delirium symptoms faster than the placebo, may result in less time with hallucinations, inattention and symptom fluctuation and may allow patients to spend more time at a desirable level of consciousness. Future studies evaluating pharmacologic therapy for ICU patients with delirium or subsyndromal delirium may benefit from monitoring the resolution and duration of individual delirium symptoms and long-term cognitive function to determine whether the different psychiatric symptoms of delirium are associated with worse or better outcomes.

## Key messages

• This study, the first to measure the response of individual delirium symptoms to antipsychotic therapy in the ICU, demonstrates that quetiapine use may be associated with more rapid resolution of many common ICU delirium symptoms compared to placebo, resulting in less time affected by hallucinations, inattention, symptom fluctuation and an undesirable level of consciousness.

• Psychomotor agitation and hyperactivity, a potential marker for lower mortality among delirious patients but predictive of worse outcome among patients not delirious, took longer to resolve with quetiapine in this pilot study, but quetiapine appears to lead to more time without agitation or depressed consciousness.

• Future studies evaluating antipsychotics in ICU patients with delirium should measure the resolution of individual delirium symptoms.

## Abbreviations

APACHE II: Acute Physiology and Chronic Health Evaluation II; DSM: diagnostic and statistical manual; ICDSC: intensive care delirium screening checklist; ICU: intensive care unit; IQR: interquartile range.

## Competing interests

The authors declare that they have no competing interests. None of the authors have conflicts of interest surrounding this study.

This study was presented in part at the 40^th ^Annual Congress of the Society of Critical Care Medicine, January, 2011.

## Authors' contributions

JWD was responsible for the concept, acquisition and interpretation of data, manuscript preparation, and final manuscript approval. YS, RRR, NH and EH were responsible for the acquisition and interpretation of data, manuscript preparation, and final manuscript approval. RR, RJR, JJF and EG were responsible for the interpretation of data, manuscript preparation, and final manuscript approval.
